# Biocontrol Agents Reduce Progression and Mycotoxin Production of *Fusarium graminearum* in Spikelets and Straws of Wheat

**DOI:** 10.3390/toxins13090597

**Published:** 2021-08-27

**Authors:** Lucile Pellan, Cheikh Ahmeth Tidiane Dieye, Noël Durand, Angélique Fontana, Sabine Schorr-Galindo, Caroline Strub

**Affiliations:** 1 Qualisud, Univ Montpellier, Avignon Université, CIRAD, Institut Agro, IRD, Université de La Réunion, F-97490 Montpellier, France; cheikh.dieye@umontpellier.fr (C.A.T.D.); noel.durand@cirad.fr (N.D.); angelique.fontana@umontpellier.fr (A.F.); sabine.galindo@umontpellier.fr (S.S.-G.); 2 CIRAD, UMR Qualisud, F-34398 Montpellier, France

**Keywords:** microorganism interactions, DON, ddPCR, biological control

## Abstract

The aim of this study was to evaluate the interactions between wheat plant (spikelets and straws), a strain of mycotoxigenic pathogen *Fusarium graminearum* and commercial biocontrol agents (BCAs). The ability of BCAs to colonize plant tissue and inhibit the pathogen or its toxin production was observed throughout two phases of the life cycle of pathogens in natural conditions (colonization and survival). All evaluated BCAs showed effective reduction capacities of pathogenic traits. During establishment and the expansion stage, BCAs provoked an external growth reduction of *F. graminearum* (77–93% over the whole kinetic studied) and mycotoxin production (98–100% over the whole kinetic studied). Internal growth of pathogen was assessed with digital droplet polymerase chain reaction (ddPCR) and showed a very strong reduction in the colonization of the internal tissues of the spikelet due to the presence of BCAs (98% on average). During the survival stage, BCAs prevented the formation of conservation perithecia of the pathogen on wheat straw (between 88 and 98% of perithecia number reduction) and showed contrasting actions on the ascospores they contain, or perithecia production (−95% on average) during survival form. The mechanisms involved in these different interactions between *F. graminearum* and BCAs on plant matrices at different stages of the pathogen’s life cycle were based on a reduction of toxins, nutritional and/or spatial competition, or production of anti-microbial compounds.

## 1. Introduction

In agricultural production, some fungal pathogens, such as certain *Fusarium* strains, produce mycotoxins, such as trichothecenes, that contaminate food and can cause health problems, such as vomiting, immunotoxic effects, or reproductive disorders [1]. In 2019 in Central Europe, almost 65% of the food and feed samples analyzed contained mycotoxins above threshold limits [2]. Cereals and cereal based-products are the first factor of consumer exposure to mycotoxins [3], especially deoxynivalenol (DON). Moreover, mycotoxins are difficult to degrade and are very stable during transformation processes [4].

Fighting against these fungal pathogens is mainly based on the massive use of chemical pesticides pre-harvest with a risk of acute or chronic toxic effects and a danger to environmental ecosystems [5]. The use of antagonistic microorganisms (Biocontrol Agents, BCAs) able to colonize and prevent the progression of pathogens in crops by various mechanisms, including parasitism, competition, or production of antifungal compounds [6], could be an alternative strategy for a more integrative and health-conscious for the environment and consumers [6,7].

To develop this approach, one of the most devastating diseases of wheat, Fusarium head blight (FHB), caused mainly by *F. graminearum*, a mycotoxigenic fungus, was used as a model. *F. graminearum* is the dominant species of the FHB complex in many European countries [8], leading to yield and quality losses of cereal production, but also to contamination by some trichothecenes [3,9,10]. At the beginning of field epidemy and when environmental conditions are favorable, *F. graminearum* infects wheat plants at the flowering of the ears, during the formation of anthers. After spore germination, the mycelium colonizes the tissues via wounds or natural openings. After this first phase, which is described as biotrophic, the fungus triggers tissue necrosis by accumulation of mycotoxins and intracellular growth, allowing it to progress rapidly to invade other close spikelets. The production of mycotoxins is essential for the successful colonization of wheat ears by *F. graminearum*, and its synthesis is stimulated by the plant’s defense compounds [11]. The life cycle of the pathogen then continues after the wheat harvest in the crop debris with saprophytic activity until the formation of the conservation structure, the perithecia. The pathogen can survive in this form under unfavorable conditions for a long period of time, leading to infected crop residues being the main potential source of inoculum [12].

Dual confrontation assays, usually used to study interactions between mycotoxigenic pathogens and biocontrol agents, are not really representative of the complex tripartite interaction between the pathogen, the BCAs, and the plant [13]. Studies carried out in the field with *F. graminearum* are subject to numerous environmental factors that could make it impossible to precisely identify the effect of biocontrol agents on the growth and mycotoxin production of this pathogen. To better understand the potential capabilities of BCAs, the use of detached organs represents a realistic interaction easier performed than field tests [14]. This study focused on key stages of interaction between the life cycle of wheat and *F. graminearum* (establishment, colonization, and survival) to analyze the impact of BCAs on this patho-system at different levels, from whole organ to gene. During the vegetative and the survival stages, either on detached spikelets or on wheat straw, the effects of BCAs on colonization, mycotoxin production or perithecia formation by *F. graminearum* were investigated using complementary techniques, including microscopy, microbiology, biochemistry, and molecular biology. This research allowed to depict behavior of three contrasting BCAs: one Actinobacteria from the *Streptomyces* genus (abbreviated Myco), one fungus from the *Trichoderma* genus (abbreviated Xeda) and one oomycete from the *Pythium* genus (abbreviated Poly), in interaction with the mycotoxigenic phytopathogen.

## 2. Results

Results of antagonistic activities of BCAs against *F. graminearum* was presented, following keys stages of interaction between *F. graminearum* and the wheat life cycle.

### 2.1. Wheat Spikelet Colonization by Microorganisms

**Figure 1:**
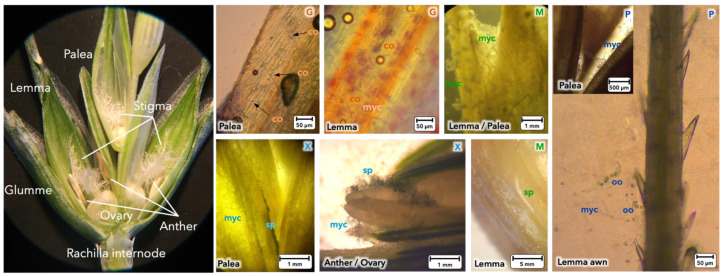
Establishment of the different microorganisms on intern tissues of wheat spikelet. Bold letters: **G**, *F. graminearum* (orange); **M**: Myco, Mycostop^®^ (green); **X**: Xeda, Xedavir^®^ (turquoise blue); **P**: Poly, Polyversum^®^ (dark blue). Lower case letters indicate the different part of microorganisms: myc: mycelium, sp: sporulation, co: *F. graminearum* conidia, oo: Poly oogonia. Optical microscope images were taken on spikelets with only the pathogen or BCAs after 8 days of growth on spikelet at 20 °C with photoperiod.

First of all, detached spikelets were inoculated with each microorganism separately. After 8 days of incubation, spikelets were dissected and internal tissues were observed to verify the endophyte capacities of the pathogen and BCAs. In Figure 1, optical microscope images show the different constituent parts of wheat spikelet (awn, lemma, palea, anther, ovary) and colonization of microorganisms in situ. *F. graminearum* (Figure 1G), rapidly colonized the palea/lemma and was able to penetrate into the tissue via stomata and to produce large quantities of conidia. All three BCAs were able to colonize the internal tissues of the spikelet. Myco (Figure 1M) colonized the epidermis of the tissues and produced numerous small pellet colonies, specific to actinomycetes. A characteristic white spore layer was produced on the surface. Xeda (Figure 1X) invaded the tissues with aerial mycelium. It used damaged areas at the edge of palea tissues, or more fragile tissues, such as the ovary inside the spikelet, to produce sporulating colonies. Poly (Figure 1P) was also able to settle and colonize the palea or the base of the lemma awn and quickly produced a large quantity of characteristic oogonia containing oospores. None of the BCAs inoculated alone caused any apparent symptoms on the spikelets (no loss of chlorophyll, necrosis, or desiccation) as compared to those inoculated with *F. graminearum* alone.

### 2.2. Inhibition of F. graminearum External Colonization during Antagonist Assays in Spikelet

**Figure 2:**
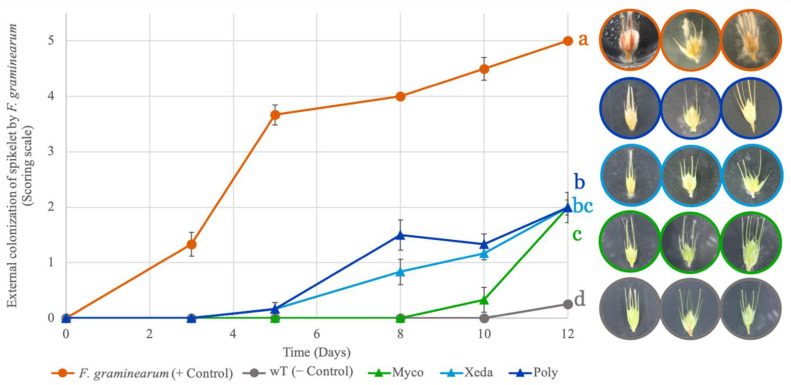
Comparative effects of BCA treatments on the external colonization of spikelets by *F. graminearum* during antagonist bioassay. Colors of curve and photography outline correspond to different treatment: Myco: Mycostop^®^, Xeda: Xedavir^®^, Poly: Polyversum^®^ in confrontation with *F. graminearum*. The pictures illustrate the level of contamination after 10 days of growth of pathogen and BCAs. Antagonist bioassays were performed on detached spikelet for 12 days at 20 °C with photoperiod. External colonization was expressed using scoring scale (from level 0, assigned level 5 for spikelets strongly infected with mycelium, necrosis and possible presence of sporodochia), described in Experimental Section (modified from [14]). Data table is presented in the Supplementary Information (Table S1) and 15 replicates were considered. Graphic presents means and SE. ANOVA tests were conducted on area under growth curves, *p*-value < 0.05.

Antagonist bioassays between *F. graminearum* and the BCAs were performed on detached spikelets and the external colonization of spikelets by *F. graminearum* was assessed using a scoring scale (from 0 to 5), to characterize the visible progression of the disease. Two clearly separate profiles of *F. graminearum* apparent development were observed between positive control with the pathogen alone and during BCAs treatment (Figure 2). *F. graminearum* alone clearly invaded spikelets tissues and produced high quantities of mycelium, pigment, and spores. BCAs treatment provoked two significative important levels of inhibition with 77% and 93% reductions of external colonization of *F. graminearum* by Poly and Myco, respectively. Xeda provided intermediate protection with 82% of *F. graminearum* visible development inhibition. Over 12 days, the wT negative control spikelets started to dry out, making it impossible to conduct the experiment on living spikelets any longer.

### 2.3. Quantification of F. graminearum Inhibition and Xeda–Pathogen Interaction in Internal Wheat Spikelet Tissues

**Figure 3:**
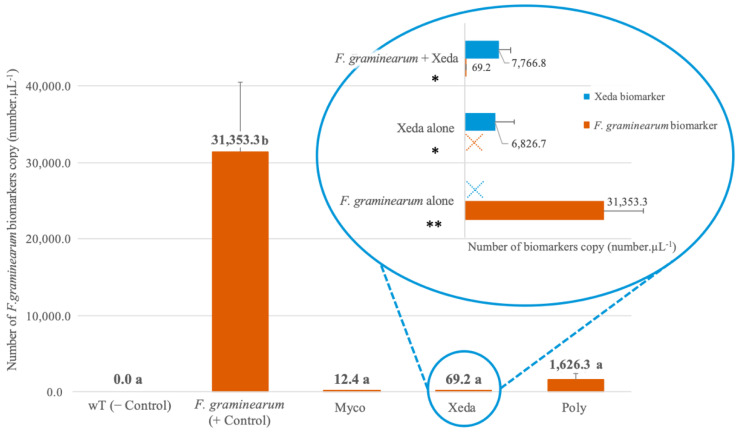
Comparative quantification of *F. graminearum* and Xeda biomarkers on detached spikelet. Antagonist bioassays were performed on detached spikelet at 20 °C with a photoperiod, and analyzed after 8 days of co-culture. Main graphic presents quantity of *F. graminearum* biomarkers in number of copy·μL^−1^ in presence of different BCAs treatments. Myco: Mycostop^®^, Xeda: Xedavir^®^, Poly: Polyversum^®^. A focus was realized on *F. graminearum*–Xeda interaction in blue circle: quantity of *F. graminearum* biomarkers copy and Xeda biomarkers. * in number of copy·μL^−1^ was compared alone or in confrontation on detached spikelet. ** For both graphic, number of biomarkers copy·μL^−1^ correspond to μL of ddPCR reaction mixture (or 22 μL by sample as described in the Experimental Section). Data table is presented in the Supplementary Information (Table S2). Three replicates were considered, graphic present means and SD. ANOVA test, *p*-value < 0.01.

To complete the external assessment of *F. graminearum* on detached spikelets, absolute quantification of this fungi was made in all confrontation conditions via ddPCR. After 8 days of growth, the quantity of *F. graminearum* biomarker copies (*Tri5*) was assessed, and important inhibitions caused by BCAs treatment could be observed (Figure 3). All BCAs triggered a quasi-complete disappearance of pathogen, similar to wT control, with 99.96%, 99.78%, and 94.81% of DNA copies detected, respectively, for Myco, Xeda, and Poly treatment. A focus was conducted on the *F. graminearum*–Xeda interaction because Xeda showed an interesting inhibition profile. It presented an intermediate decrease of the pathogen on the visual growth scale, which allows to detect it but also to detect the pathogen; and a total inhibition of mycotoxins, which is a decisive advantage in the potential valorization of cereal production. The copy number of the Xeda biomarker (rpb2) was analyzed on detached spikelets inoculated either with this agent alone, with the pathogen alone, or during the *F. graminearum*–Xeda interaction. Under the conditions where the microorganisms are alone, there was no nonspecific amplification detected with the two biomarkers used. While the colonization capacity of the pathogen alone represents 100% colonization, this BCA has a colonization capacity of 20% and therefore does not completely invade the plant tissue. The colonization levels of Xeda were significantly similar whether they were inoculated alone or with *F. graminearum*. During this *F. graminearum*–Xeda interaction, the detection of the pathogen was strongly reduced (99.78% growth inhibition), illustrating the efficiency of colonization and pathogen inhibition by this BCA.

### 2.4. Inhibition of DON Production by F. graminearum during Antagonist Assay in Spikelet

**Figure 4:**
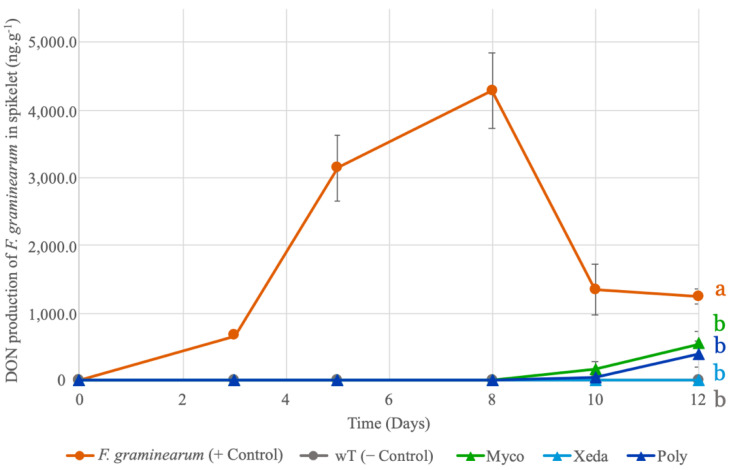
Comparative effects of BCA treatments on DON production of *F. graminearum* in spikelet during antagonistic bioassay. Colors of curve correspond to different treatment: Myco: Mycostop^®^, Xeda: Xedavir^®^, Poly: Polyversum^®^ in confrontation with *F. graminearum*. Antagonistic bioassays were performed on detached spikelet for 12 days at 20 °C with photoperiod. DON production was expressed in ng·g^−1^ of spikelet. Data table is presented in the Supplementary Information (Table S1) and 12 replicates were considered. Graphic presents means and SE. ANOVA tests were conducted on area under mycotoxin production curves, *p*-value < 0.01.

A method for extraction and quantification of trichothecene produced by *F. graminearum* in unitary wheat spikelets samples were optimized and used to monitor the evolution of mycotoxins production during antagonist bioassays with BCAs. Although the *F. graminearum* strain used in this study has the ability to produce all three mycotoxins (DON, 15-AcDON, 3-cADON) in vitro, in a previous study, in the spikelet antagonistic assays, only DON was detected by the method used and 15-AcDON, 3-AcDON were below limits of detection. The results presented in Figure 4 shows a bell-like evolution with the detection of DON as early as 3 days of infection and peak of mycotoxin production between 5 and 8 days of *F. graminearum* positive control samples. This peak production is completely avoided in samples treated separately using the three types of BCAs (*p*-value < 0.01), which showed total inhibition of DON production until day 8. Xeda, in particular, maintained this inhibition throughout the observed kinetics.

### 2.5. BCAs Impact on Perithecia Synthesis and Quantity on Wheat Straw

**Figure 5:**
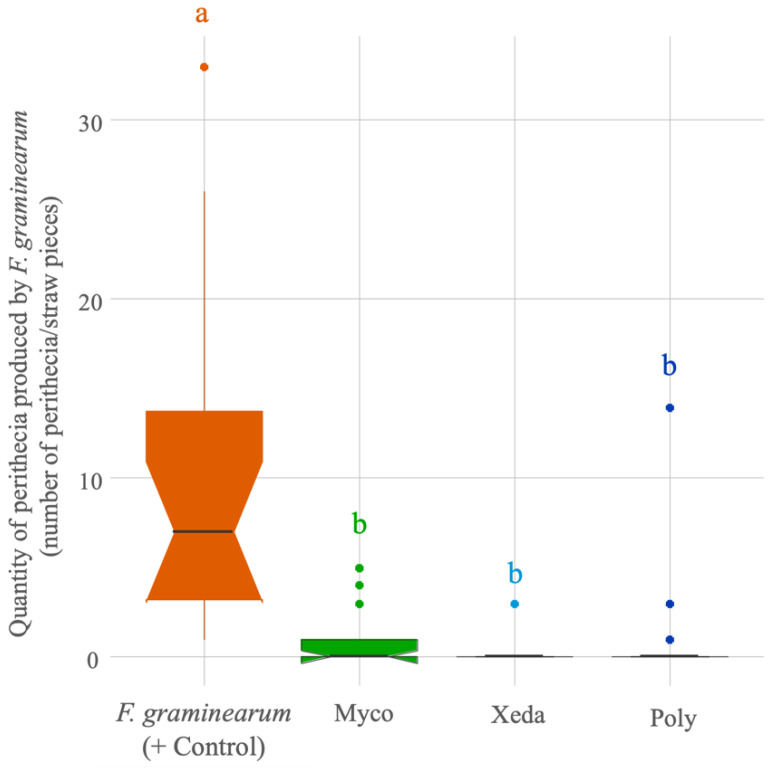
Comparative effect of BCAs on perithecia production by *F. graminearum* during antagonistic bioassay on wheat straw. Myco: Mycostop^®^, Xeda: Xedavir^®^, Poly: Polyversum^®^ in confrontation with *F. graminearum*. Antagonistic bioassays were performed on wheat straw for 3 weeks at 20 °C in dark. Boxplot represents the distribution of the 18 replicates per conditions, ANOVA test, *p*-value < 0.05. Data table is presented in the Supplementary Information (Table S3).

A selected method used for antagonist assays on wheat straws permits to decipher the impact of BCAs on the production of perithecia, the survival form of *F. graminearum* inter season. After three weeks of incubation with a high humidity, *F. graminearum* formed many dark perithecia (ten per wheat straw on average, but which could be up to 30 perithecia per wheat straw) as can be seen in Figure 5. On the contrary, pieces treated with BCAs presented a very strong inhibition of perithecia formation by the pathogen. Xeda caused the strongest effect with almost a 99% reduction, and Myco and Poly resulted in an average 90% reduction in the quantity of perithecia. In spite of this strong inhibition, some samples treated with Poly could, nevertheless, allow the development of these conservation structures with up to 14 perithecia at most.

**Figure 6:**
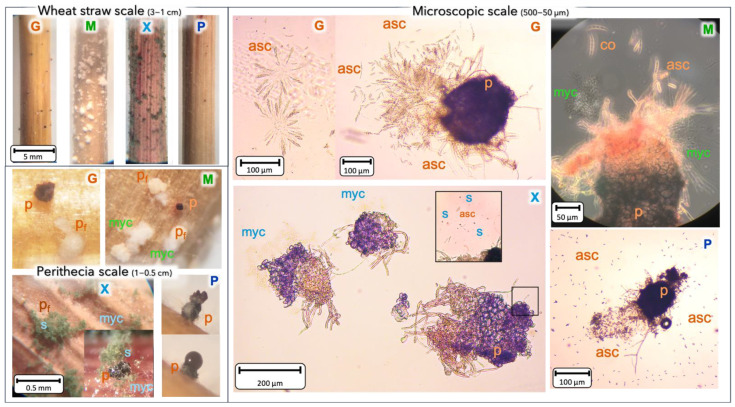
Macroscopic and microscopic BCAs impact on perithecia produced by *F. graminearum*. Pictures show the interaction between *F. graminearum* perithecia and the three BCAs at different levels in situ or on microscope slides. Bold letters: **G**, *F. graminearum* (orange); **M:** Mycostop^®^ (green); **X**: Xedavir^®^ (turquoise blue); **P**: Polyversum^®^ (dark blue). Lower case letters indicate the different parts of microorganisms: myc: mycelium, sp: sporulation, asc: *F. graminearum* ascospores, co: *F. graminearum* conidia, pf: perithecia in formation, *p*: perithecia. Optical microscope images were taken after 3 weeks of incubation on wheat straw at 20 °C.

Once the perithecia were counted, the wheat straw was observed under a microscope at different scales and was photographed (in situ or on microscope slide) in order to highlight the particular modes of action of the BCAs when interacting with the pathogen perithecia (Figure 6). In the first scale (1–3 cm), perithecia appeared on all positive control samples with only *F. graminearum*. Important quantity of characteristic colonies of Myco and Xeda were spread along the pieces of wheat. Poly presented very low visible mycelial growth and only few perithecia were produced by *F. graminearum* compared to the control. Increasing the magnification to the scale of the perithecia (0.5–1 cm), the control samples showed both developing and mature perithecia. In confrontation with Myco, the BCA colonies developed in contact with emerging perithecia and the few mature perithecia were reduced in size. In a dual bioassay against Xeda, perithecia in formation were covered with Xeda sporulating mycelium. Some mature perithecia were directly parasitized by BCA colonies. The perithecia formed during the interaction between *F. graminearum* and Poly all showed abnormalities, such as early excretion of ascospores in cirrhus, external wall deformations, and droplet exudations. The perithecia of the control samples contained large quantities of ascospores with a particular star-shaped organization where each branch was composed of imbricate ascospores. This spore cluster conformation could not be observed on any perithecia that had been treated with BCAs. Myco and Xeda prevented rigidification of parts of the perithecia wall (lighter areas), and very few ascospores of the pathogen could be observed when the perithecia were burst. Colonies of these BCAs could be observed on the perithecia fragments, confirming the presence of BCAs onside perithecia. Samples treated with Myco also showed the presence of conidia of *F. graminearum*. Xeda produced many visible spores when observed close to the perithecia residues. In a completely different way, the perithecia deformed by the action of Poly produced numerous disorganized ascospores in a mucilage inside the perithecia. These ascospores were very small compared to those produced in the controls (respectively, 15 and 25 µm on average).

## 3. Discussion

Experiments characterizing the impact of BCAs on *F. graminearum* have been carried out through the phases of the natural interaction between the life cycles of wheat and the pathogen (establishment, colonization and survival of pathogen). This chronological continuity allows to propose ways to improve the use of BCAs and to identify the best application stages during field epidemic [15] to make them more competitive than conventional agrochemicals. The antagonistic bioassays on detached spikelets were analyzed at different levels (visual notation, DNA quantification and mycotoxin production). The use of spikelets compared to the whole plant allows to test a large quantity of biotic and abiotic conditions from a small area of wheat growing, while approaching the natural matrices on which the interaction takes place in the field. This step can help identify the most promising BCA candidates prior to the whole plant study. Indeed, during the study of the screening of different BCAs against *F. graminearum* on detached spikelet, a divergence was observed between the results obtained during the duals culture tests on synthetic medium and thus obtained on spikelets [14] while correlations could be observed between tests on detached spikelet and greenhouse assays on whole plants in another study [16].

The BCAs studied were analyzed in terms of reduction of growth and mycotoxin production in dual culture assays on synthetic media during a previous study [17] and significant differences were observed with the results obtained in situ on detached spikelets. Concerning the pathogen, the global mycotoxin maximum level was reduced by one third in plant tissues and the chemotype has completely switched from 15-ADON produced mainly in vitro with a small amount of DON, to exclusively DON produced on spikelets. Usually in field studies, DON was the most common type B trichothecenes [18,19], which underlines the importance of identifying appropriate tests, closer to natural infection conditions, for a reliable interpretation of the mode of interaction. Regarding the antagonistic activities of BCAs on *F. graminearum*, they had all been underestimated in in vitro dual assays to varying degrees [17]. Myco, which had a very low level of inhibition of *F. graminearum* in vitro, showed a strong inhibition in detached spikelet. With intermediate profile, Xeda allowed strong inhibition of visual growth and mycotoxin inhibition in situ and medium visual growth and mycotoxin inhibition in dual culture assays. Finally, *F. graminearum* inhibition caused by Poly was best estimated in dual culture assays than in detached spikelet. This difference between the two types of tests were similar that which already highlighted in another study [14].

In the first days of colonization of the spikelets by the pathogen, limited mycelium was visible but discoloration of the spikelet tissues began to appear, suggesting that the pathogen was possibly in the biotrophic phase and was colonizing the tissues without apparent classical symptoms. The important mycotoxin production at the beginning of analysis (<5 days) tended to decrease as the pathogen entered in a second phase which appears to be saprophytic phase, while the mycelium developed abundantly [11,20,21]. These results are consistent with a comparative study of the colonization of *F. graminearum* on live and dead wheat ears [11], which indicated that a majority of the pathogen genes used the plant tissue as a signal for the induction of mycotoxin biosynthesis.

Concerning the test of microorganism establishment and growth on spikelets, good tissue colonization was observed and no BCAs caused symptoms when inoculated alone on the spikelets. Xeda mainly accumulated on already weakened areas (as cut rachilla internode), suggesting symbiotic or saprophytic activity [22]. Several *Trichoderma* spp. have already shown features including opportunistic and avirulent plant symbionts that cause substantial changes in host metabolism, favoring growth, and disease resistance [23].

The assessment of *F. graminearum* colonization and disease development in plant tissues is frequently characterized using a scoring scale [24,25,26]. Correlation was found between reduced symptom expression and reduced biomass of *F. graminearum* [16]. In our study, all BCAs showed a significant reduction in the external development of *F. graminearum* infection, assessed using visual scoring scale. Xeda and Myco that particularly developed on spikelets, could be good competitor against pathogen. Indeed, these two BCAs compared to *F. graminearum* alone showed good nutritional competition capacity on synthetic medium in a previous study, and Xeda only a good space competition capacity [17]. It is therefore relevant to point out that Myco is also spatially competitive on spikelets. Nevertheless, visual distinction between BCA colonization and pathogen may be difficult depending on the BCA considered. Moreover, describing the infection only by extern visual scoring of the spikelets does not allow to know precisely the internal development of the pathogen, even by adapting the scoring scale by taking into account the signs of early colonization by this pathogen (level 1: discoloration of wheat tissues). To overcome this problem, a method for the detection and quantification of microorganisms, via ddPCR, was developed. This technique has already been successfully used to analyze the presence of other pathogens in plant tissues [27] and has the advantage of being a highly precise method for sensitive DNA detection and absolute quantification. Moreover, ddPCR is less susceptible than qPCR to substances that inhibit PCR reactions and favorize reproducibility and specificity of assays [28]. This method can therefore allow a specific and early detection of pathogen, confirming a definite advantage in the case of pathogens with similar appearance or which symptoms appear late in the colonization process, such as *F. graminearum*. To our knowledge, this is the first report of *F. graminearum* quantification on wheat via this method. By comparing the results of visual scores and quantification, similar trends can be observed (same order of effectiveness of the three BCAs: Control > Poly > Xeda > Myco). However, external observation alone overestimated the relative presence of *F. graminearum* in the samples treated with BCAs compared to the control (84% versus 98% inhibition on average recorded during visual scoring or absolute quantification of *F. graminearum*). In previous tests, Xeda caught our attention because it showed strong inhibition of pathogen growth (but not complete allowing potential pathogen detection), and total inhibition of mycotoxin production. Xeda biomarker was also detected. This is why a focus was made on the interaction between this BCA and the pathogen, and this assay demonstrated that Xeda was able to colonize spikelet but also to drastically reduce the presence of *F. graminearum*. Surprisingly, its growth capacity did not seem to be affected by the presence of *F. graminearum* during interaction, which contributed to its efficiency during field epidemics [16]. This innovative method of detection (ddPCR coupled with specific primers) permits a precise quantification of pathogen and BCAs co-inoculated on wheat tissues. It opens up an interesting path for future studies on BCAs colonization and biocontrol competition against phytopathogens.

This pathogen growth analysis can be compared to mycotoxin production results to characterize an overall protective effect of BCAs. The method developed for the precise quantification of mycotoxins allows to observe significant differences between the evolution of visual and/or absolute growth of pathogen (ddPCR) and its capacities to produce mycotoxin. This toxin is mainly produced during the primary phase of the biotrophic mode of pathogen [29], which is coherent with our results. All the BCAs studied were very effective in the reduction of mycotoxins accumulation in detached spikelets in this first phase (<5 days) and even beyond (<10 days); in particular Xeda, which induced a complete inhibition of the DON produced by *F. graminearum*. This may be due to many mechanisms, such as inhibition of pathogen growth, biotransformation of the mycotoxins produced [30] or epigenetic changes caused by BCAs that prevent pathogenic Fusarium pathogens by inhibiting the expression of genes of mycotoxin biosynthesis pathways [31], but these activities remain complex. The small increase of mycotoxins at the end of the kinetics could be explained by a lower activity of BCAs during the saprophytic phase of the pathogen, but the presence of BCAs has greatly reduced the amount of toxins detected below the limit recommended by the European Union for DON in unprocessed durum wheat: 1750 µg·kg^−1^ (Commission Regulation (EC) No 1881/2006, [32]).

During this first phase, all BCAs demonstrate potentialities for the reduction of colonization and mycotoxins production of *F. graminearum*. As suggested by the analyses on the modes of action of these BCAs in vitro, competition, mycophagy or mycotoxin bio-transformation phenomena could be responsible for the success of these BCAs [17,33]. The mechanisms implemented by these BCAs during in vitro tests could be investigated more precisely in order to prove their deployment in natural conditions.

It is well described that conservation structures, such as perithecia, preserved in culture debris are the primary source of inoculum for future epidemics [34]. The activity of BCAs against perithecia of *F. graminearum* was then evaluated. These results are consistent with those obtained in vitro on Carrot agar in another study (80% inhibition on average, [33]. All BCAs showed a substantial reduction in the number of mature perithecia; however, the mechanisms involved in this reduction seem to vary according to the BCA considered. Indeed, Myco and Xeda seemed to enter directly into spatial competition with *F. graminearum* and parasitize the perithecia. It seemed to cause a weakening of the structure of the perithecia and a strong reduction in the quantity of ascospores. This effect may be due to the direct synthesis of antimicrobial compounds that will interact with the formation of these structures [35]. On the contrary, Poly will allow more perithecia to reach maturity, and will potentially provoke an increase in the number of ascospores, but with atrophied structures. Straws colonized by Myco or Xeda have a worse appearance than the positive control straws; however, it is important to highlight that colonization of wheat straw by those BCAs was higher than colonization by *F. graminearum* when these two types of microorganisms were inoculated. In field conditions, crop debris are not consumed as food or feed and the straw remains in the soil. Nevertheless, further studies could be done on the persistence of these BCAs in soil, and their impact on microbial communities, although, in general, BCAs do not disturb the populations present [36,37,38]. The BCAs could even participate in the degradation of the straw, which would reduce the substrates for the formation of perithecia by *F. graminearum* [39].

## 4. Conclusions

Based on this integrative analysis, all the commercial BCAs showed effective reduction capacities in both phases of the *F. graminearum* cycle (vegetative and survival stages), whereas their recommended commercial use is limited to aerial parts (Poly) or for general soil treatments (Myco and Xeda). Future efforts to improve BCA disease control on wheat spikes and in the phyllosphere of various plants should focus on application timing, which could be extended throughout the pathogen cycle in case of commercial BCAs or tested on this different stage for new potential BCAs.

BCAs are able to act at different levels of the pathogen’s life cycle but also through different mechanisms such as the reduction of toxins, nutritional and/or spatial competition, or production of anti-microbial compounds. They are therefore an effective means of limiting the use of pesticides through integrated management strategies, while maintaining a level of food safety of production. The proposed model on spikelets allows to have some quickly and easily answers on the mode of interaction of microorganisms closer to real situations thanks to this complex matrix. Moreover, ddPCR method is a rapid and reliable solution to quantify the pathogen and BCAs development during this interaction. The developed protocols can be used to observe other patho-systems, only with the pathogen for the assessment of resistant varieties or abiotic factors, or in biocontrol studies with pathogen and other agents or natural substances.

## 5. Materials and Methods

### 5.1. Micro-Organisms and Culture Condition

*F. graminearum* isolate BRFM 1967 was chosen for its isolation origin (wheat plant) and its strong ability to produce mycotoxins (CIRM, University of Aix-Marseille, Marseille, France) with a deoxynivalenol/15-Acetyldeoxynivalenol/3-Acetyldeoxynivalenol (DON/15-AcDON/3-AcDON) chemotype. Fungus were maintained under paraffin oil at 4 °C and actively grown on PDA (PDA; BD Difco, Sparks, MA, USA) at 25 °C for 7 days for spore production. Spore suspension was prepared from solid medium in sterile water-Tween (wT, 0.01%) and filtered with carded cotton.

Three commercial biological control agents were selected because of their contrasting types and uses: Mycostop^®^ (*Streptomyces griseoviridis*, Lallemand Plant Care^®^), Xedavir^®^ (*Trichoderma asperellum*, Xeda International^®^), and Polyversum^®^ (*Pythium oligandrum* DeSangosse^®^). Explanations on how BCAs were selected and more information about their characteristics and their effects on growth and mycotoxinogenesis of *F. graminearum* in dual culture assays are available in previous work [17]. All BCAs were isolated from their commercial formulation with a classical microbial isolation protocol and conserved in commercial product aliquots (4 °C) or in glycerol solution (15%/V:V/−80 °C). Pure cultures were maintained on ISP4 (agar 18 g; starch 10 g; K_2_HPO_4_·3H_2_O 1 g; MgSO_4_·7H_2_O 1 g; (NH_4_)_2_SO_4_ 1 g; CaCO_3_ 1 g; FeSO_4_·7H_2_O 0.001 g; MgCl_2_ 0.001 g; ZnSO_4_·7H2O 0.001 g; per liter of distilled water), PDA, and V8 juice agar (V8 juice 200 mL (Campbell’s, Camden, NJ, USA); agar 15 g; CaCO_3_ 3 g; per 800 mL of distilled water), respectively, for Mycostop, Xedavir, and Polyversum at 25 °C for 7 days for production of spore suspensions or filaments for Poly. Spores suspension were prepared as described for *F. graminearum*. For this study, the strain isolated from commercial product were referred using the following abbreviations: M or Myco for Mycostop (Lallemand Plant Care^®^, Castelmaurou, France), X or Xeda for Xedavir (Xeda International^®^, Saint-Andiol, France), and P or Poly for Polyversum (DeSangosse^®^, Pont-du-Casse, France).

**Figure 7:**
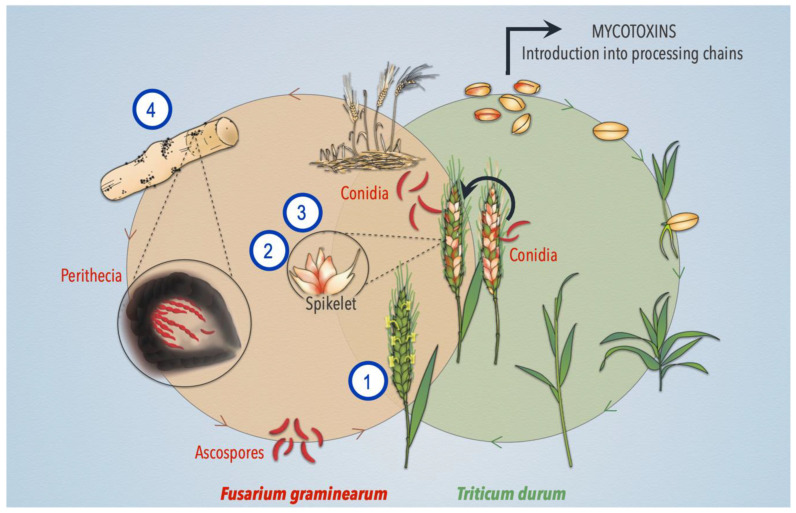
*Fusarium graminearum* and *Triticum durum* life cycles with a focus on their interconnection stages, and position of different assays carried out in this study. (**1**) Inoculation and establishment of microorganisms at the flowering stage of spikelets in accordance to the natural attack phases of *F. graminearum*, (**2**) monitoring of external growth of *F. graminearum* via a visual scoring scale and internal assessment via DNA quantification of microorganisms on the spikelet, (**3**) analysis of DON production by *F. graminearum* on spikelets, and (**4**) monitoring of BCAs effect on the survival structures of the pathogen, the perithecia.

Antagonistic activities of BCAs against *F. graminearum* was assessed at keys stages of interaction between *F. graminearum* and wheat life cycle, as presented in Figure 7: (1) Inoculation of microorganisms at the flowering stage of wheat in relation to the natural attack phases of *F. graminearum*, (2) monitoring of external growth via a visual scoring scale and internal assessment via specific DNA quantification of the microorganisms on spikelet, (3) analysis of the production of DON on spikelets during interaction with BCAs, and finally (4) the effect of BCAs on the survival structures of the pathogen, the perithecia.

### 5.2. Antagonistic Activity of BCAs on Wheat Spikelets 

#### 5.2.1. Inoculation and Visual Growth Assessment of Wheat Spikelet

Dual culture assay with *F. graminearum* and the three BCAs were conducted on detached wheat spikelet as described by [14]. *Triticum durum* wheat, cultivar Miradoux^®^ (Florimond Desprez^®^, Cappelle-en-Pévèle, France) were grown from untreated seeds on culture room (20 °C, photoperiod 16 h day/8 h night with a 400 W sodium lamp (Phillips, Amsterdam, the Netherlands) up to the BCCH 55–60 stage (beginning of ear emergence and flowering). Spikes of the homogenized stage were harvested and spikelets were detached and used immediately for confrontation. 

Detached spikelets were dipped in spore suspension of BCAs or water-Tween (wT) control (1 × 10^5^ spores·mL^−1^) and placed in Petri dishes containing agar and water medium (3 g·L^−1^) with rachilla inserted in medium to conserve spikelets. After four days, spikelets was rapidly dipped in *F. graminearum* spore suspension (1 × 10^4^ spores·mL^−1^) or wT control and repositioned to the minimum medium. All spikelets were incubated at 20 °C (photoperiod 16 h day/8 h night). Every two days, a visual evaluation of the external colonization of *F. graminearum* was realized for each spikelet, using a visual scale modified from [14], during 12 days. Level 0 was assigned to no symptom of disease, level 1 to spikelet tissue degeneration, level 2 to beginning of necrosis apparition, level 3 to visible mycelium on all spikelet tissue, level 4 to mycelium and necrosis and level 5 to spikelets strongly infected with mycelium, necrosis and possible presence of sporodochia. For each condition and sampling days, 15 spikelets were considered. Samples inoculated with the pathogens or BCAs were dissected and intern tissues was examined using a binocular loupe and a Zeiss PrimoStar optical microscope with Zeiss Axiocam ERc5s camera (Carl Zeiss Microscopy, Thornwood, NY, USA) to verify the colonization capacities of microorganisms. The experiment was performed twice and similar results were observed.

#### 5.2.2. Quantification of BCAs-Pathogens Interaction in Wheat Spikelet Tissues by ddPCR

Three spikelets of each confrontation conditions were collected 8 days after inoculation, and stored at −80 °C for further analysis. For the DNA extraction, samples were grinded using GenoGrinder (SpexSamplePrep, Metuchen, NJ, USA) with steel grinding balls. Total DNA was extracted using NucloSpin DNA extraction kit (Macherey-Nagel, GmbH & Co.KG, Düren, Germany) according to the manufacturer’s instructions. Absolute quantification of *F. graminearum* in all conditions was realized by IAGE company (Montpellier, France). As Xeda presented an interesting inhibition profile (intermediate on growth and total inhibition of mycotoxins), it was selected to be detected in interaction with the pathogen. Detection and quantification of these two microorganisms was performed with DropletDigitalPCR (ddPCR) technique. Biomarkers primers used in this study were detailed in Table 1. They were chosen for the specific detection and quantification of *F. graminearum* and Xeda (*T. asperellum*). DNA was detected and quantified using the QX200™ Droplet Digital™ PCR system (Bio-Rad, Pleasanton, CA, USA). The ddPCR reaction mixture contained 2 μL of DNA template (22 μL total reaction volume); 450 nM of each EvaGreen primer and sterile water. Samples were partitioned with 70 μL of BioRad droplet oil in a BioRad QX200 droplet generator. Water-in-oil emulsions were transferred to a 96-well plate and amplified in a PCR thermocylcler using the following cycling protocol: hold at 95 °C for 5 min, 40 cycles of 95 °C for 30 s, 62 °C for 1 min, and a final enzyme deactivation step at 98 °C for 10 min followed by cooling at 10 °C. After the thermal cycling, the plate was then analyzed on droplet reader. Wells containing all reagents and RNA/DNA—free water were used as negative control. The software package provided with the ddPCR system (QuantaSoft v1.7, Bio-Rad) was used for data acquisition.

#### 5.2.3. DON, 15-AcDON and 3-AcDON Extraction and Quantification on Wheat Spikelet 

For mycotoxin analysis, each of twelve remaining spikelets by conditions was grinded with liquid nitrogen and 5 mL of acetonitrile/water/acetic acid (79:20:1, *v/v/v*) was added. Samples were homogenized by mechanical agitation for 20 min. Samples were preliminarily diluted 1:50 in water/acetic acid (99.5:0.5; mobile phase of analyzer) and filtered with a CA filter (0.45 µm, Carl Roth GmbH, Karlsruhe, Germany) before injection. Mycotoxin detection and quantification were achieved using an Ultra High-Performance Liquid Chromatography (UHPLC, Shimadzu, Tokyo, Japan) coupled with a mass spectrometer (8040, Shimadzu, Tokyo, Japan) as described in [17]. Mycotoxins detection and quantification were achieved using ultra high-performance liquid chromatography (UHPLC) coupled with a mass spectrometer (8040, Shimadzu). LC separation was performed using a Phenomenex Kinetex XB Column C18 (50 mm × 2 mm; 2.6 μm particles) at 50 °C, with an injection volume of 50 μL. Mobile phase composition was (A) 0.5% acetic acid in ultra-pure water and (B) 0.5% acetic acid in isopropanol (HPLC MS grade, Sigma) and mobile phase flow rate was 0.4 mL·min^−1^. The mass spectrometer was operated in electrospray positive (ESI+) and negative (ESI−) ionization mode, and two multiple reaction monitoring (MRM) transitions for each analyte were monitored for Quantification (Q) and Qualification (q) (Table 2 and Table 3). For each sample batch, one sample containing a trichothecenes standard (Type A and B Trichothecenes, TrilogyLab, Washington, MO, USA) is analyzed randomly (100 ng·mL^−1^). All data were analyzed using LabSolution Software (Shimadzu, Tokyo, Japan). During the development of this method on spikelet, various parameters were tested to ensure the reliability of the analysis. At the extraction level, the quantity of spikelet (one or three), grinding (liquid nitrogen or fresh grinding), ratios of solvent (5 mL or 15 mL) and dilution in the mobile phase (1:50, 1:10 or 1:2) have been tested and the best parameters were selected. The following points were also checked: the absence of molecules in healthy spikelet having the same transitions as the mycotoxins, the high extraction yield (84.5%) using labelled mycotoxin standards added to healthy spikelets during extraction, repeatability of the analyses carried out on varied contaminated samples by inoculation of spores, and detection even of small quantities of mycotoxins (with DON limits of detection or quantification of 4 ng·mL^−1^ and 14 ng·mL^−1^, respectively; 15-AcDON limits of detection or quantification of 10 ng·mL^−1^ and 35 ng·mL^−1^, respectively; 3-AcDON limits of detection or quantification of 15 ng·mL^−1^ and 50 ng·mL^−1^, respectively). The experiment was performed twice and similar results were observed.

#### 5.2.4. Antagonistic Activity on Wheat Straw and *F. graminearum* Perithecia Production

Dried wheat straws were collected at the end of the cycle of wheat plants used previously, to assess the antagonistic activity of BCAs against *F. graminearum* and monitoring the *F. graminearum* perithecia production (survival form) [42]. Three-centimeter-long straws were immersed in distilled water for 12 h, removed from the water, and autoclaved twice (121 °C for 15 min). The sterilized straws were then dried in sterile filter paper, dipped in *F. graminearum* wT spore suspension (1 × 10^4^ spores·mL^−1^) for 2 min and were placed in center of Petri dish with small water-soaked sterile filter discs on the periphery. After two days, BCAs spore suspensions or Control were distributed along each straw piece. Plates were incubated in culture room at 22 °C in dark with 80% of relative humidity for 3 weeks (Bioclimatic chamber ICH750 C, Memmert, Schwabach, Germany) to allow the interaction of microorganisms on residue. Eighteen replicates of each condition were made and perithecia from each condition were counted, then gently crushed between slide and lamella in order to observe their contents at microscopic scale, using a binocular loupe and a Zeiss PrimoStar optical microscope with Zeiss Axiocam ERc5s camera (Carl Zeiss Microscopy, Thornwood, NY, USA). Another method consisted in incubating wheat straws in sterile wet soil was tested to produce *F. graminearum* perithecia and gave similar results to the selected method but the counting of perithecia was difficult because of the similar soil color.

#### 5.2.5. Data Treatment and Statistical Analysis

With the aim of considering complete growth and mycotoxin production kinetics, and not only a final point, three parameters have been extracted from the curves: the area under the curve, the maximum speed and the plateau reached. The area under disease progression and mycotoxin production curve was finally chosen because it is the most discriminant parameter to differentiate several conditions. Statistical data analysis was performed with R Software (3.4.4, R Foundation for Statistical Computing, Vienna, Austria, 2017). Normality and homogeneity of variances were verified and the effect of BCA treatments or difference between conditions was tested with a one-way ANOVA and multiple comparisons of means were done with Tukey’s test (α = 0.05). Graphics program used for the artwork was Adobe Illustrator (Adobe, San Jose, CA, USA).

## Figures and Tables

**Table 1:** Primer pairs characteristics used for absolute quantification by ddPCR.

Gene	Sequence		Amplicon Size	Targeted Organisms	Source
*Tri5*	F	GATCTGATGACTACCCTCAATTCCTT	71	*F. graminearum*	[40]
	R	GCCATAGAGAAGCCCCAACAC			
RNA PolyB subII (*rpb2*)	F	GGAGGTCGTTGAGGAGTACGAA	142	Xeda (*T. asperellum*)	[41]
	R	TTGCAGATAGGATTTACGACGAGT			

**Table 2:** MS/MS parameters for isotope labelled, internal standard (**IS**) (Romer Labs, Getzersdorf, Austria).

IS	Polarity	MRM	EC
U-(^13^C_15_)-DEOXYNIVALENOL	−	370.3 > 59.0	35

**Table 3:** MS/MS parameters for mycotoxins. (**Q**) Quantification; (**q**) qualification.

	Polarity	MRM Q	EC MRM Q	MRM q	EC MRM q	R^2^ Calibration Curve
DON	−	355.0 > 59.0	35	355.0 > 265.1	35	0.9998
15-AcDON	+	338.90 > 137.10	−19	338.90 > 297.20	−14	0.9988
3-AcDON	−	397.20 > 59.00	25	397.20 > 307.10	16	0.9988

## Data Availability

The datasets generated and/or analyzed during the current study are available from the corresponding author on reasonable request.

## Supplementary Materials

The following are available online at https://www.mdpi.com/article/10.3390/toxins13090597/s1, Table S1: Comparative effects of BCA treatments on the external colonization and mycotoxin content of spikelets by *F. graminearum* during antagonist bioassay, Table S2: Comparative quantification of *F. graminearum* and Xeda biomarkers on detached spikelet, Table S3: Comparative effect of BCAs on perithecia production by *F. graminearum* during antagonistic bioassay on wheat straw.
