# Supplementary material for: Biocontrol Agents Reduce Progression and Mycotoxin Production of Fusarium graminearum in Spikelets and Straws of Wheat
Source: Toxins (Basel). 2021 Aug 27;13(9):597. doi: 10.3390/toxins13090597 (PMC8470793; doi:10.3390/toxins13090597)
Supplement: Supplementary file 1 [file toxins-13-00597-s001.zip › toxins-1292183-supplementary.pdf]

Supplementary Materials: Biocontrol Agents reduce progression and mycotoxin production of *Fusarium graminearum* in spikelets and straws of wheat

Lucile Pellan, Cheikh Ahmeth Tidiane Dieye, Noël Durand, Angélique Fontana, Sabine Schorr-Galindo and Caroline Strub

Table S1. Comparative effects of BCA treatments on the external colonization and mycotoxin content of spikelets by *F. graminearum* during antagonist bioassay. Antagonist bioassays were performed on detached spikelet for 12 days at 20°C with photoperiod. N.d.: Not detected, wT: water-Tween, 0.01%.

Conditions	Day	External Colonisation (Scoring scale)			Mycotoxin Content (DON) (ug.kg^{-1})		
		Number of Replicate	Mean	Standard Error	Number of Replicate	Mean	Standard Error
wT (- Control)	3	15	0.00	0.00	12	0.00/n.d.	0.00
F. graminearum (+ Control)	3	15	1.33	0.21	12	650.94	69.07
F. graminearum + Mycostop	3	15	0.00	0.00	12	0.00/n.d.	0.00
F. graminearum + Xedavir	3	15	0.00	0.00	12	0.00/n.d.	0.00
F. graminearum + Polyversum	3	15	0.00	0.00	12	0.00/n.d.	0.00
wT (- Control)	5	15	0.00	0.00	12	0.00/n.d.	0.00
F. graminearum (+ Control)	5	15	3.67	0.21	12	3046.22	675.40
F. graminearum + Mycostop	5	15	0.00	0.00	12	0.00/n.d.	0.00
F. graminearum + Xedavir	5	15	0.17	0.17	12	0.00/n.d.	0.00
F. graminearum + Polyversum	5	15	0.17	0.17	12	0.00/n.d.	0.00
wT (- Control)	8	15	0.00	0.00	12	0.00/n.d.	0.00
F. graminearum (+ Control)	8	15	4.00	0.00	12	4307.23	1046.60
F. graminearum + Mycostop	8	15	0.00	0.00	12	0.00/n.d.	0.00
F. graminearum + Xedavir	8	15	0.83	0.17	12	0.00/n.d.	0.00
F. graminearum + Polyversum	8	15	1.50	0.22	12	0.00/n.d.	0.00
wT (- Control)	10	15	0.00	0.00	12	0.00/n.d.	0.00
F. graminearum (+ Control)	10	15	4.50	0.22	12	1337.56	581.21
F. graminearum + Mycostop	10	15	0.33	0.33	12	164.10	164.10
F. graminearum + Xedavir	10	15	1.17	0.17	12	0.00/n.d.	0.00
F. graminearum + Polyversum	10	15	1.33	0.21	12	46.04	46.04
wT (- Control)	12	15	0.33	0.21	12	0.00/n.d.	0.00
F. graminearum (+ Control)	12	15	5.00	0.00	12	1240.86	145.70
F. graminearum + Mycostop	12	15	2.00	0.37	12	543.29	230.16
F. graminearum + Xedavir	12	15	2.00	0.26	12	0.00/n.d.	0.00
F. graminearum + Polyversum	12	15	2.00	0.37	12	384.48	288.24

Table S2. Comparative quantification of *F. graminearum* and Xeda biomarkers on detached spikelet. Antagonist bioassays were performed on detached spikelet at 20°C with photoperiod, and analyzed after 8 days of co-culture. wT: water-Tween, 0.01%.

**Conditions**	**Number of Replicate**	**Quantity of biomarker copy of *F. graminearum* (nbr.µl^{-1})**		**Quantity of Biomarker Copy of Xedavir (nbr.µl^{-1})**	
		**Mean**	**Standard Deviation**	**Mean**	**Standard Deviation**
wT (- Control)	3	0.00	0.00	0.00	0.00
F. graminearum (+ Control)	3	31353.33	9085.75	0.00	0.00
Mycostop	3	0.00	0.00	0.00	0.00
F. graminearum + Mycostop	3	12.38	5.37	0.00	0.00
Xedavir	3	0.00	0.00	6826.67	4381.58
F. graminearum + Xedavir	3	69.15	51.83	7766.83	2612.58
Polyversum	3	0.00	0.00	0.00	0.00
F. graminearum + Polyversum	3	1626.33	766.12	0.00	0.00

Table S3. Comparative effect of BCAs on perithecia production by *F. graminearum* during antagonistic bioassay on wheat straw. Antagonistic bioassays were performed on wheat straw for 3 weeks at 20°C in dark.

**Conditions**	**Number of Replicate**	**Quantity of Perithecia (nb. of perithecia)**	
		**Mean**	**Standard Error**
F. graminearum (Control)	18	10.39	2.22
F. graminearum + Mycostop	18	1.17	0.42
F. graminearum + Xedavir	18	0.17	0.17
F. graminearum + Polyversum	18	1.06	0.78
